# Deep learning for gradability classification of handheld, non-mydriatic retinal images

**DOI:** 10.1038/s41598-021-89027-4

**Published:** 2021-05-04

**Authors:** Paul Nderitu, Joan M. Nunez do Rio, Rajna Rasheed, Rajiv Raman, Ramachandran Rajalakshmi, Christos Bergeles, Sobha Sivaprasad, for the SMART India Study Group, Pramod Bhende, Rajiv Raman, Ramachandran Rajalakshmi, Viswanathan Mohan, Kim Ramasamy, Taraprasad Das, Padmaja K. Rani, Rupak Roy, Supita Das, Deepa Mohan, V. Narendran, George Manayath, Giridhar Anantharaman, Mahesh Gopalakrishnan, Sundaram Natarajan, Radhika Krishnan, Sheena Liz Mani, Manisha Agarwal, Tapas Padhi, Umesh Behera, Harsha Bhattacharjee, Manabjyoti Barman, Gajendra Chawla, Alok Sen, Moneesh Saxena, Asim K. Sil, Subhratanu Chakabarty, Thomas Cherian, K. R. Reesha, Rushikesh Naigaonkar, Abishek Desai, Col Madan Deshpande, Sucheta Kulkarni, Dolores Conroy, Jitendra Pal Thethi, Radha Ramakrishnan, Janani Surya

**Affiliations:** 1 Institute of Ophthalmology, University College London, London, EC1V 9EL UK; 2 Section of Ophthalmology, King’s College London, London, WC2R 2LS UK; 3 Retina Department, Vision Research Foundation, Sankara Nethralaya, Chennai, Tamil Nadu India; 4 Dr. Mohan’s Diabetes Specialities Centre and Madras Diabetes Research Foundation, Chennai, Tamil Nadu India; 5 School of Biomedical Engineering and Imaging Sciences, King’s College London, London, SE1 7EU UK; 6 NIHR Moorfields Biomedical Research Centre, Moorfields Eye Hospital, London, EC1V 2PD UK; 7 Dr. Mohan’s Diabetes Specialities Centre, Chennai, Tamil Nadu India; 8 Aravind Eye Hospital, Madurai, Tamil Nadu India; 9 LV Prasad Eye Institute, Hyderabad, Telangana India; 10 Sankara Nethralaya, Kolkata, India; 11 Dr Mohan’s Diabetes Specialities Centre, Bangalore, Karnataka India; 12 Aravind Eye Hospital, Coimbatore, Tamil Nadu India; 13 Giridhar Eye Institute, Cochin, Kerala India; 14 Aditya Jyot Hospital, Mumbai, Maharashtra India; 15 Dr Tony Fernandez Eye Hospital, Aluva, Kerala India; 16 Dr Shroff’s Charity Eye Hospital, New Delhi, India; 17 LV Prasad Eye Institute, Bhubaneshwar, Odisha India; 18 Sri Sankaradeva Nethralaya, Gawahati, Assam India; 19 Vision Care Clinic & Research Centre in Arera Colony, Bhopal, Madhya Pradesh India; 20 Sadguru Netra Chikitsalaya, Chitrakoot, Madhya Pradesh India; 21 Aurobindo Nethralaya, Raipur, Chhattisgarh India; 22 Netra Niramay Niketan, Haldia, West Bengal India; 23 Little Flower Hospital & Research Center, Angamaly, Kerala India; 24 Ganapathy Nethralaya, Jalna, Maharashtra India; 25 HV Desai Hospital, Pune, Maharashtra India; 26 UCL Institute of Ophthalmology, London, UK; 27 B005 Meenakshi Classic, Bangalore, India; 28 UCL Institute of Ophthalmology, London, UK; 29 Retina Department, Vision Research Foundation, Sankara Nethralaya, Chennai, India

**Keywords:** Retinal diseases, Medical imaging, Population screening

## Abstract

Screening effectively identifies patients at risk of sight-threatening diabetic retinopathy (STDR) when retinal images are captured through dilated pupils. Pharmacological mydriasis is not logistically feasible in non-clinical, community DR screening, where acquiring gradable retinal images using handheld devices exhibits high technical failure rates, reducing STDR detection. Deep learning (DL) based gradability predictions at acquisition could prompt device operators to recapture insufficient quality images, increasing gradable image proportions and consequently STDR detection. Non-mydriatic retinal images were captured as part of SMART India, a cross-sectional, multi-site, community-based, house-to-house DR screening study between August 2018 and December 2019 using the Zeiss Visuscout 100 handheld camera. From 18,277 patient eyes (40,126 images), 16,170 patient eyes (35,319 images) were eligible and 3261 retinal images (1490 patient eyes) were sampled then labelled by two ophthalmologists. Compact DL model area under the receiver operator characteristic curve was 0.93 (0.01) following five-fold cross-validation. Compact DL model agreement (Kappa) were 0.58, 0.69 and 0.69 for high specificity, balanced sensitivity/specificity and high sensitivity operating points compared to an inter-grader agreement of 0.59. Compact DL gradability model performance was favourable compared to ophthalmologists. Compact DL models can effectively classify non-mydriatic, handheld retinal image gradability with potential applications within community-based DR screening.

## Introduction

Recent advances in portable retinal camera technology and telemedicine have made remote, low-cost ophthalmic screening viable^1^. Diabetic retinopathy (DR) affects one in three of the 463 million people living with diabetes worldwide and is the leading cause of acquired vision loss in economically active adults^2,3^. However, with access to DR screening, early identification and treatment of sight-threatening DR (STDR) can reduce the risk of visual loss by over 50%^4^. Desktop-based, mydriatic retinal imaging with DR severity grading by trained staff is an effective but resource intensive screening strategy^1,4^. Therefore, there is an upsurge of DR screening using handheld retinal photography without pharmacological dilation in the community or opportunistically in non-clinical environments in low- and middle-income countries (LMIC), as a viable, low-cost option relative to desktop, mydriatic retinal imaging^1,5^. Handheld, non-mydriatic retinal imaging, combined with advances in deep learning (DL) assisted STDR detection^5–7^, could significantly expand viable DR screening availability in communities with limited healthcare access^2^, notably amongst LMIC^8^.

The capture of retinal images using handheld retinal cameras without pupil dilation, poses specific challenges^2,4,9^. Handheld systems do not have a stabilising platform and are therefore prone to image blur at acquisition. Retinal imaging may also be more difficult in communities with limited healthcare access due an increased prevalence of undiagnosed co-pathologies. The presence of cataract and diabetes associated pupil miosis^10^ can negatively affect image quality^1,9^. The proportion of gradable images using handheld retinal cameras without mydriasis is reported to be 70–76% compared to 90% with dilation^8,11^. However, capturing gradable quality retinal images is critically important to achieving the recommended minimum STDR detection sensitivity (80%) and specificity (95%) required for a clinically effective screening^5,12,13^. The inclusion of the optic disc within retinal images is also important as optic disc neovascularisation is significantly associated with visual loss^14^.

Given the portability of handheld retinal cameras and the negligible costs of multi-image acquisition per patient, on-device automated image gradability classification and feedback to field operators would support the recapture of insufficient quality images, in turn maximising the proportion of gradable images. Gradability classification systems would also be useful in research for the automated labelling of large retinal image datasets. Previous approaches for automated gradability classification required a number of pre-processing steps including image attribute extraction^15–19^, retinal component detection (e.g. fovea or vessels)^20,21^, retinal component segmentation (e.g. vasculature)^22–25^ or reference derivation^26^. These pre-requisites complicate the implementation of such systems on low-cost, mobile and handheld retinal imaging devices. DL is advantageous as no explicit image feature selection is required and models for use within mobile and processing limited devices are readily available^27,28^. Prior DL gradability classification models were trained on largely mydriatic, retinal image datasets captured on desktop cameras, hence are not well-suited to datasets from non-mydriatic, portable devices^6,7,29,30^.

The aim of this study is to evaluate whether DL models can learn to classify the gradability of handheld, 2-field non-mydriatic retinal images. We created a representative sample of retinal images captured as part of a community-based, house-to-house DR screening study distributed over 20 rural sites in India. We trained a computationally efficient, compact gradability classification DL model suitable for low-cost, mobile and handheld retina imaging devices using ophthalmologist derived ground-truth labels. We compared compact DL model predictions to ophthalmologist labels and reviewed model performance at three operating points contrasted with inter-grader metrics. Finally, we contrasted compact DL model performance to a larger model on the gradability task.

## Methods

The study is approved by the Indian Council of Medical Research (ICMR)/Health Ministry Screening Committee (HMSC). The study was conducted in accordance with the tenets of the Declaration of Helsinki. Each patient provided informed consent for participation in the study. The ORNATE India project is a 4-year Global Challenge Research Fund (GCRF) and UK Research and Innovation (UKRI) funded multicentre study whose ambition is to build research capacity and capability to tackle DR related visual impairment in India and the UK^31^. One key aim is to initiate community-based DR screening in India using a low-cost, non-mydriatic portable camera (SMART India study) and train DL models to assist in the automated detection of DR^31^. One of the first steps to achieving this goal is the development of an image quality assessment tool that can assist device operators in the acquisition of gradable retinal images^31^.

### Study design, setting and participants

Anonymised retinal images used in this cross-sectional study were captured as part of the SMART India study between 21 August 2018 and 30 December 2019. There were 20 active sites distributed in 13 states and 1 union territory around India where community-based, house-to-house DR screening was performed in people with known diabetes or a random blood sugar 160 mg/dL (≥ 8.9 mmol/L) on the day of screening^31^. The included sites were Aluva, Angamaly, Bangalore, Bhopal, Bhubaneswar, Chennai (*2 sites*), Chitrakoot, Cochin, Coimbatore, Guwahati, Haldia, Hyderabad, Jalna, Kolkata, Madurai, Mumbai, New Delhi, Pune and Raipur. All field operators from the 20 sites were trained on the steps for optimal fundal image capture and the use of the handheld Zeiss Visuscout 100 retinal camera (Germany) by Zeiss personnel. Each field operator practised and was observed capturing at least 10 images prior to deployment. Zeiss personnel also provided additional, intensive, one-week training to help field operators consolidate their fundal image capture skills. A set of fovea-centred and optic disc centred images were captured by trained field operators from each eye without the application of mydriatic agents using the handheld retinal camera. Patients in whom the acquisition of retinal images was not possible, potentially due to small pupils or cataracts, had photographs of the anterior segment taken with the same camera. Images were labelled as left or right by field operators. For each patient eye, a variable number of images were captured. The group of images from each eye were graded collectively by up to two SMART India graders (optometrists or ophthalmologists) independently with eyes labelled as gradable or ungradable; however, there were no gradability labels for individual images. The group of images from each eye were also assessed in aggregate for DR severity with senior ophthalmologist arbitration when there were disagreements between graders. Patients also had several self-reported characteristics recorded at the community screening visit including age, gender, smoking history, diabetic status, presence of significant cataract or history of cataract surgery in either eye^31^.

### Dataset curation

**Figure 1:**
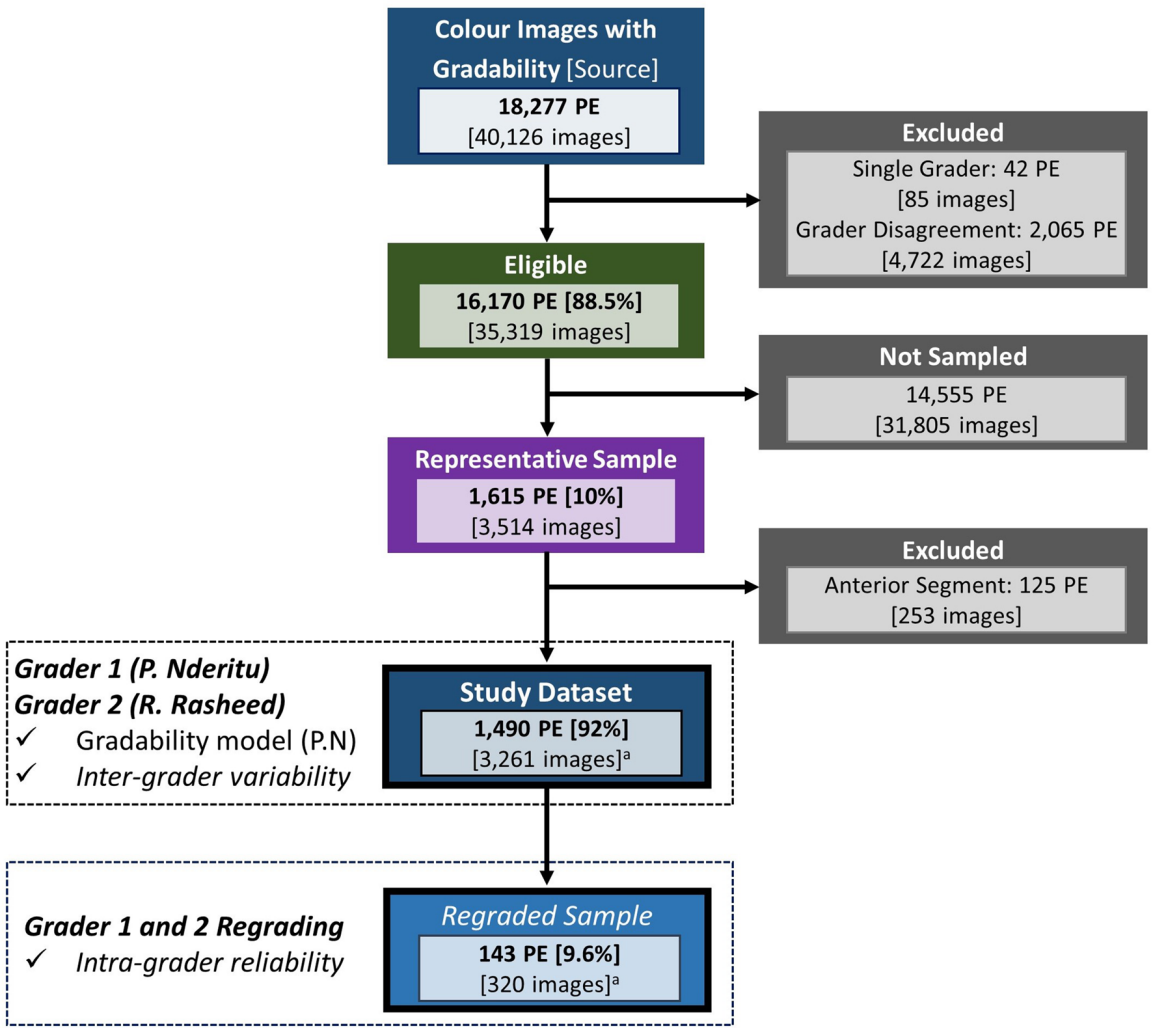
Data curation, sampling and study dataset construction. *PE* Patient eyes, ^a^All images per patient eye were graded.

The source dataset consisted of colour images from patient eyes of known gradability and laterality. Patient eyes with two gradability labels with agreement between SMART India graders were eligible (Fig. 1).

#### Sampling

As the proportions of site, DR grade and gradability varied within eligible patient eyes, stratified sampling with proportional allocation was used to derive a representative sample dataset of patient eyes. Strata consisted of sites (20), DR grades (no DR grade, non-referable DR, referable DR) and SMART India gradability (gradable, ungradable). There were 90 strata with 10% of patient eyes randomly sampled from each. The chosen sampling proportion would yield sufficient retinal images for DL model training based on prior studies^29,30^.

### Gradability definition

A simple, pragmatic definition of gradability was used to maximise consistency and repeatability. The optic disc and retinal vessels were used as key landmarks for the application of the gradability definition. The complete capture of the optic disc was important to ensure cases of neovascularisation were not missed given the significantly increased risk of sight loss if left untreated^14^. A study defined gradable fovea-centred image implies that the majority of the captured macula was gradable.

**Figure 2:**
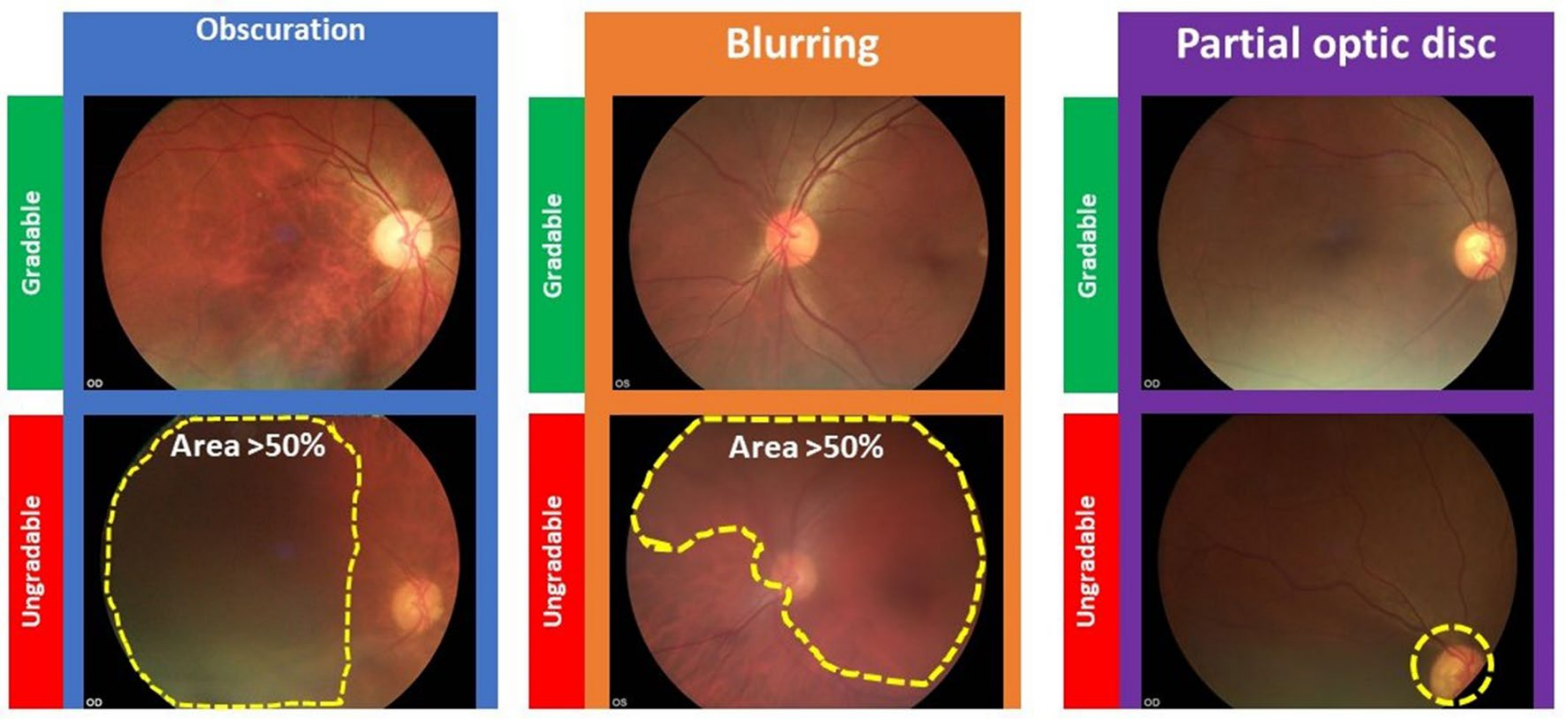
Gradability definition examples. *OD* Right eye, *OS*: Left eye.

Images were considered gradable if all of the following were true (Fig. 2):

1. Less than 50% of the image area is obscured or over/under exposed (*judged using retinal vessels*)
2. Less than 50% of the image area is blurred or out of focus (*judged using retinal vessels*)
3. The whole optic disc is captured within the image (*notably for fovea-centred images*)

### Ophthalmologist grading

Both grader 1 (P. Nderitu) and grader 2 (R. Rasheed) are experienced ophthalmology fellows trained in conducting retinal research and DR grading. Grader 1 evaluated sampled images and excluded non-retinal (anterior segment) images with the remaining retinal images per patient eye graded by both graders. As there were multiple images from the same eye, the order of images was randomised prior to grading to reduce bias from assessing sequential, potentially correlated retinal images. Images from ~ 10% of study patient eyes, selected using stratified, proportional sampling, were regraded one week later to estimate intra-grader reliability.

### Model development

#### Pre-processing

Images were resized to 224 × 224 × 3 from their native resolution (1536 × 1152 × 3) by nearest neighbour interpolation, chosen for its simplicity and efficiency. The conservative input size kept computational requirements low to align with the capabilities of portable retinal imaging systems. Left eye images were horizontally flipped to a right orientation to reduce inter-image variance. During model training, images were augmented by applying random brightness (± 20%), zoom (+ 25%), vertical flip and rotations (± 5 degrees) sequentially with an occurrence probability of 0.5 which produced plausible physiological images.

#### Compact model architecture

The EfficientNet^28^ model family have recently been demonstrated to achieve state-of the-art accuracy and efficiency with 6 × faster inference speed at 8 × smaller computational cost and with good transfer capability compared to previous deep convolutional neural network architectures^28^. With the potential application of gradability models on handheld or mobile devices, computational efficiency and accuracy were key considerations hence the use of EfficientNet-B0 as the primary, compact base model for this study. EfficientNet-B0 has incorporated rescaling (0–1) and per channel normalisation layers $((x-\mu )/\sigma )$, where $x$ is the input pixel value, μ is the image brightness mean value and $\sigma$ is the standard deviation value with ‘ImageNet’ based per channel (red, green, blue) mean and standard deviation constants (0.485, 0.456, 0.406 and 0.229, 0.224, 0.225)^28^. The base model was connected to a classification model that consisted of a 3 × 3 depth-wise separable 2D convolution (16 kernels, stride 1, ‘swish’ activation^32^) layer followed by batch normalisation^33^ and dropout (0.5)^34^. These layers were repeated but with 32 kernels in the next depth-wise separable convolution layer. Finally, the classification model output was reshaped into a flattened array which was adjoined to a single output node with sigmoid activation (Supplementary Fig. 1). Both depth-wise separable convolutions and dense layers had L2 regularisation (kernel and bias, c = 0.01). Depth-wise separable convolution layers were employed as they are more computationally efficient relative to standard convolutional operations but with retained accuracy^27^. There were ~ 4.08 million parameters (42,119 untrainable) in the final model whose total size was 48 MB.

#### Large model architecture

To contrast the difference in performance if a larger model was used for gradability classification, we selected the large EfficientNet-B5 variant (~ 28.5 million parameters, total size 327 MB) as a base model but kept the classification model architecture constant (Supplementary Fig. 2).

#### Training methodology

Both EfficientNet models were pre-trained on ‘ImageNet’, with the pretrained weights used for initialisation^28^. The compact DL model was trained on a single Intel i7-8700k CPU, with the larger EfficientNetB5 model trained on a single GPU (Nvidia Quadro P6000); models were developed using Tensorflow (v2.1). Stochastic gradient descent (Adam optimiser) with a batch size of 16 was used to minimise the binary cross-entropy loss. Proportional class weights, $(\frac{1}{x}\times total)/2.0$, where $x$ is the count of positive or negative cases, were applied during model training given the class imbalance (gradable 4:1 ungradable). Training was performed in two steps to preserve pre-trained base model weights during initial classification model training. In the first stage, the classification model alone was trained with a starting learning rate of 1e−3 which decayed exponentially after 3 epochs. In the second stage, both the classification and base models were trained with a starting learning rate of 1e−5 which reduced exponentially after 3 epochs. Batch normalisation layers were kept unchanged in both stages. Models were trained for a maximum of 20 epochs per fold to prevent overfitting with the highest validation area under the receiver operating characteristic curve (AUC-ROC) model saved after each epoch.

### Main outcomes and measures

#### Model performance

Model performance was evaluated using random, group stratified, fivefold cross validation. Images from the same patient were either in the training or testing set (but not both) given the co-correlation between eyes of the same patient. The performance of each fold was evaluated using per fold AUC-ROC and area under precision-recall curve (AUC-PR). The mean/standard deviation of the AUC-ROC and AUC-PR were estimated from the fivefolds.

To explore and compare model performance at potential operating points (OPs), different thresholds $t \epsilon \left[\mathrm{0,1}\right]$ on the classification scores provided by the model were explored:

1. OP1: $$t_{op1} = 0.5$$.
2. OP2: $t_{op2} = arg max_{t} J\left( t \right)$, where $J\left( t \right) = \left[ {sensitivity\left( t \right) + specificity\left( t \right) - 1} \right]$ is Youden’s function^35^.
3. OP3: $t_{op3} = arg min_{t} K\left( t \right)$, where $K\left( t \right) = \left[ {abs\left( {precision\left( t \right) - recall\left( t \right)} \right)} \right]$ is used to minimize unbalanced precision-recall performance.

Binary model predictions were compared to grader 1 labels as the gold standard and gradability proportions, precision, recall and Cohen’s Kappa^36^ are reported.

#### Grader performance

Grader 1 and 2 gradability proportions and inter and intra-grader agreement (Cohen’s Kappa^36^) are reported and contrasted to the model performance metrics. All statistical analyses were performed on SPSS v26 (IBM).

## Results

A total of 16,170 patient eyes (88.5%, 35,319 images) were eligible (see Fig. 1). The representative 10% sample consisted of 1615 patient eyes containing 3514 images, from which 253 non-retinal images were excluded; no patient eyes had both retinal and anterior segment images. The remaining 3261 retinal images from 1490 patient eyes (1431 patients) formed the study dataset. The mean age (years) of study patients was 56 [standard deviation (SD): 11], 52% were female and the proportion of left eyes was 50.5%. The presence of a significant cataract in one eye was reported in 8%, 51.5% were known diabetics and 4–5% had referable DR (Table 1).

**Table 1:** Study dataset patient demographics and characteristics.

Variable		N (%) or mean (SD)
Age	Years	*56 (11)*
Gender^a^	Male	685 (48.0)
	Female	743 (52.0)
Eye	Right	708 (49.5)
	Left	723 (50.5)
Smoking status^b^	Non-smoker	1,289 (90.1)
	Current or former smoker	141 (9.9)
Diabetic status^b^ (Self-reported)	Unsure	363 (25.4)
	No	330 (23.1)
	Yes	737 (51.5)
HbA1C^c^	*%*	*7.8 (2.2)*
Significant cataract in either eye	Yes	112 (7.8)
Cataract surgery in either eye	Yes	124 (8.7)
Right eye DR grade	No DR grade^d^	24 (3.4)
	Non-referable DR^e^	651 (91.9)
	Referable DR^f^	33 (4.7)
Left eye DR Grade	No DR grade^d^	93 (12.9)
	Non-referable DR^e^	600 (83.0)
	Referable DR^f^	30 (4.1)

*SD* Standard deviation, *DR* Diabetic retinopathy.

^a^3 missing gender values.

^b^1 missing value for smoking and diabetic status respectively.

^c^23 missing HbA1C values.

^d^Patient eyes with ungradable images only hence there is no DR grade.

^e^Includes no DR, mild DR and stable treated proliferative DR.

^f^Includes moderate non-proliferative DR, severe non-proliferative DR and proliferative DR.

### Main outcomes and measures

#### Compact model performance

**Figure 3:**
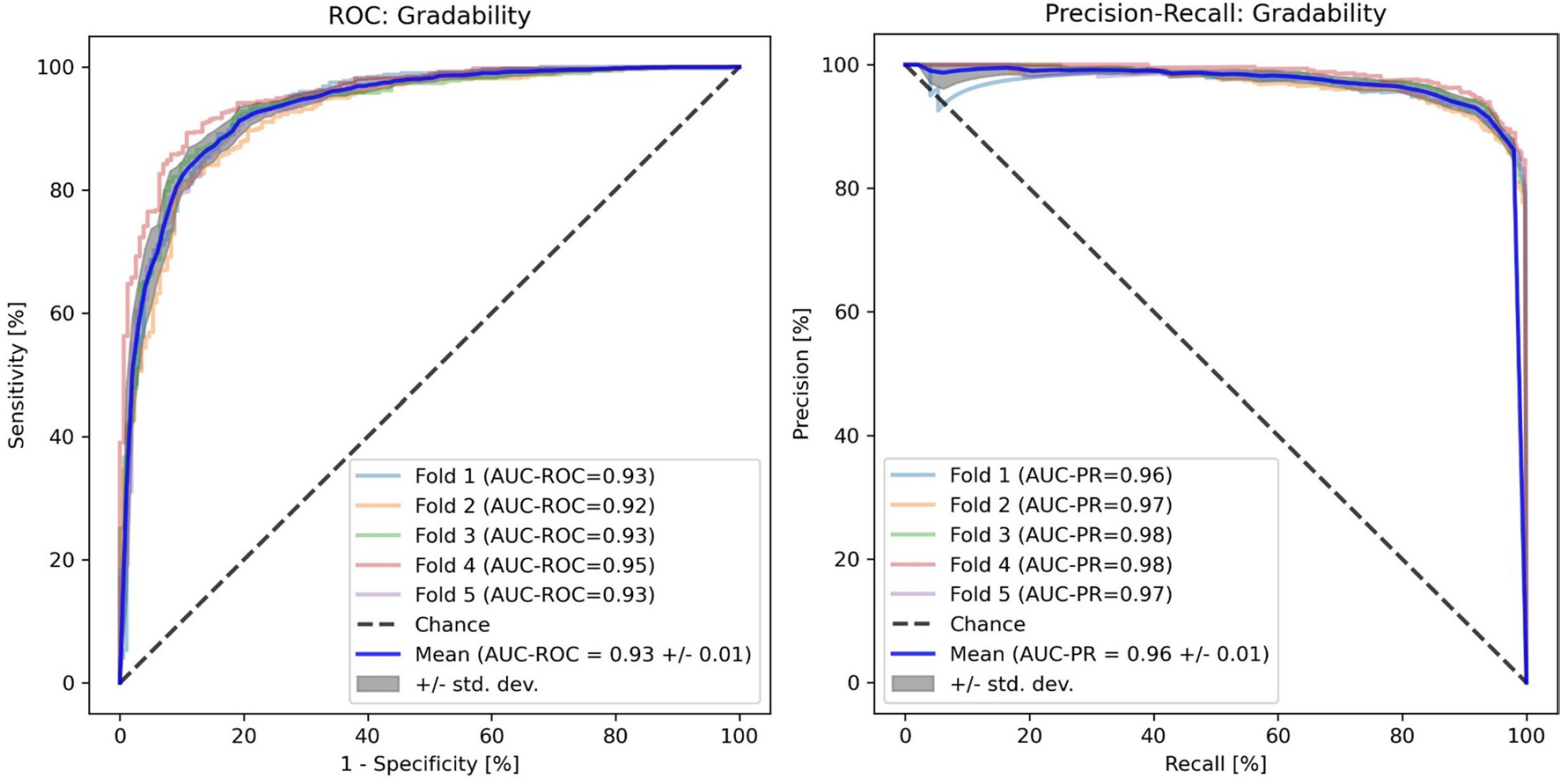
Compact Model (EfficientNet-B0) Gradability ROC and PR Curves. *ROC* Receiver operating characteristic, *AUC-ROC* Area under the receiver operating characteristic curve, *AUC-PR* Area under the precision recall curve, *std. dev* Standard deviation.

The mean (SD) AUC-ROC and AUC-PR for the compact EfficientNet-B0 model compared to grader 1 were 0.93 (0.01) and 0.96 (0.01) respectively with an AUC-ROC range of 0.92–0.95 between folds (Fig. 3). At OP1, EfficientNet-B0 model gradable precision was 0.97 with a recall of 0.77. Gradable precision was 0.94 and recall 0.89 at OP2 and equal (0.92) at OP3. Kappa values were 0.58 (0.01), 0.69 (0.01) and 0.69 (0.02) for OP1, OP2 and OP3 indicating moderate (OP1) and substantial (OP2 and OP3) agreement^36^ as shown in Table 2. The compact EfficientNet-B0 model inference time for a single retinal image gradability prediction was 38 ms.

**Table 2:** Compact model (EfficientNet-B0) and grader performance.

	OP Threshold	Gradability	Grader 1		Total N (%)	Precision [Recall]	Kappa (SE)
			Ungradable N (%)	Gradable N (%)			
Efficient Net-B0 Model	OP1 0.5	Ungradable	759 (23.3)	554 (17.0)	1313 (40.3)	0.58 [0.92]	0.58
		Gradable	66 (2.0)	1882 (57.7)	1948 (59.7)	0.97 [0.77]	(0.01)
	OP2 0.23	Ungradable	686 (21.0)	267 (8.2)	953 (29.2)	0.71 [0.88]	0.69
		Gradable	139 (4.3)	2169 (66.5)	2308 (70.8)	0.94 [0.89]	(0.01)
	OP3 0.15	Ungradable	640 (19.6)	195 (6.0)	835 (25.6)	0.77 [0.78]	0.69
		Gradable	185 (5.7)	2241 (68.7)	2426 (74.4)	0.92 [0.92]	(0.02)
Grader 2	N/A	Ungradable	544 (16.7)	215 (6.6)	759 (23.3)	0.72 [0.66]	0.59
		Gradable	281 (8.6)	2221 (68.1)	2502 (76.7)	0.89 [0.91]	(0.02)
Total N (%)			825 (25.3)	2436 (74.7)	3261 (100)	N/A	

*OP* Operating point, *SE* Standard error.

#### Large model performance

The large EfficientNet-B5 model had an AUC-ROC and AUC-PR of 0.95 (0.01) and 0.97 (0.001) respectively (Supplementary Fig. 3). EfficientNet-B5 precision, recall were 0.96, 0.86 at OP1/2 and 0.93, 0.93 at OP3 respectively. EfficientNet-B5 kappa values (0.69, 0.73) indicated there was substantial agreement at all operating points^36^ (Supplementary Table 1. EfficientNet-B5 model inference time for a single retinal image gradability prediction was 74 ms.

#### Grader performance

Within the study dataset, the proportion of gradable images was 74.7% (grader 1) and 76.7% (grader 2). Compared to grader 1, gradable precision for grader 2 was 0.89 with a recall of 0.91. There was moderate agreement between graders with a Kappa of 0.59 (0.02) (Table 2). Within the regraded sample, the proportion of gradable images was 75.3% and 78.8% for grader 1 and 2 respectively. Intra-grader reliability was substantial, Kappa 0.78 (0.04), for grader 1 and almost perfect, Kappa 0.94 (0.02), for grader 2.

## Discussion

Handheld, non-mydriatic retinal imaging has the potential to expand the deliverability of DR screening but capturing gradable quality images can be challenging^1,2,4^. Retinal image quality is fundamental to the success of community DR screening. Poor quality images may lead to erroneous false negatives, especially in DL assisted DR classification^6,7^, which limits STDR detection^13^, and increases avoidable referrals. In this study, we demonstrate a computationally efficient, compact DL model for gradability classification of handheld, non-mydriatic images that include the optic disc. Such a system could be used at the point of capture to motivate the acquisition of gradable quality retinal images to maximise STDR detection within low-cost, efficient, community-based DR screening.

Previous DL gradability models were trained on 1-field, fovea-centred retinal images acquired with the use of mydriatic agents on desktop cameras^6,29,30^. Despite this, compact EfficientNet-B0 model performance was comparable to DL-based approaches reported by Wagner et al., (AUC-PR 0.96)^29^, Pérez et al., (AUC-ROC 0.96)^30^ and Gulshan et al., (AUC-ROC 0.98)^6^. Other approaches for automated retinal image quality classification reported an AUC-ROC of 0.89^22^, 0.91^19^, 0.95^17^, 0.95^18^, 0.98^23^. However, there were significant variations in populations, methodologies (non-DL), pre-processing (extracted features), image quality definitions and acquisition (1-field, desktop retinal imaging with mydriasis) between studies^17–19,22,23^. The compact EfficientNet-B0 model achieved a gradability agreement of 0.69 compared to grader 1 at OP2 and OP3. The level of agreement at these OPs was higher than between graders (0.59). Compact DL model to grader agreement, at OP2 and OP3, was also higher than reported in previous automated image quality evaluation studies (0.64)^16,21^. Therefore, the performance of the compact EfficientNet-B0 model compares favourably to previous studies and ophthalmologist grader performance. Advantageously, the compact model was trained on 2-field retinal images, required minimal pre-processing and had modest computational resources, making it suitable for mobile and portable retinal imaging systems^27,28^. In contrast, the larger EfficientNet-B5 model showed only a marginal increase in performance (mean AUC-ROC + 0.02 and AUC-PR + 0.01) but at the cost of a significant increase in the number of parameters (~ × 6) and inference time (~ × 2) compared to the compact EfficientNet-B0 model.

In the clinical context, retinal images normally undergo a ‘gradability’ check, with gradable images selected for DR classification by human graders, and increasingly, automated systems^5–7^. Therefore, reducing the misclassification of ungradable images as gradable (maximising specificity) would be a priority to reduce the selection of poor quality images. Suboptimal quality images can increase errors in DR severity classification by human graders or automated DR grading, resulting in missed ‘positive’ DR cases. However, this requirement should be balanced with the minimisation of false rejections of gradable images as ungradable (maximising sensitivity). In the context of community DR screening, patients with no gradable images from either eye need to be referred to hospital to rule out the presence of DR using other means (*e.g. by slit-lamp examination*)^9^. Therefore, if the DL systems gradable threshold is such that a significant proportion of images are misclassified as ‘ungradable’, then field operators may be unable to capture a ‘gradable’ image from either eye despite multiple attempts, resulting in an unnecessary referral to the hospital eye service. Erroneous hospital eye service referrals would, in turn, compromise the efficiency of community DR screening programmes. This is especially relevant in community settings using hand-held retinal imaging, where image quality is affected by challenging image acquisition conditions and patient co-pathology (e.g. cataracts)^1,10^. In light of these competing objectives, we compared three OPs; OP1 with a high specificity, OP2 with a balanced sensitivity/specificity and OP3 with a high sensitivity. OP choice would vary depending on the patient population, operational factors, gradable image proportions and DR screening programme requirements. In this study, OP2 best balanced the competing requirements of maximising specificity and sensitivity.

Prior non-mydriatic retinal imaging studies have reported gradable proportions of 90% are achievable with an estimated ~ 60–70% of ungradable images due to technical failure^8,11^. Attainable gradable proportions were estimated using the compact DL model identification of ungradable retinal images at OP2 given a 70% technical failure rate. At OP2, 88% of ungradable images were correctly identified by the compact model, if 70% of these images were successfully recaptured (assuming technical failure), the proportion of ungradable images would decrease by 62%. Concurrently, the proportion of gradable images would increase to 90% and the proportion of patient eyes containing ungradable images alone would decrease from 13.4 to 2.3%. The 11% decrease in ungradable patient eyes would result in a proportional reduction in potentially avoidable hospital eye service referrals for dilated retinal examinations.

The proportion of gradable and ungradable images reported by grader 1 (74.7%) and grader 2 (76.7%) were concordant with prior studies with similar patient characteristics^8,11^. The inter-grader agreement was comparable to studies evaluating handheld, non-mydriatic (0.64) and non-handheld retinal images (0.58, 0.64) amongst ophthalmic retinal specialist graders^11,17,21^. One prior study reported higher inter-grader agreement (0.83), but this study had variable intra-grader reliability (0.48 and 0.85) and a higher proportion of ungradable images, which can significantly influence the Kappa statistic^8,36^.

Study strengths are the representative sample dataset derived from a large, community-based DR screening programme where handheld, non-mydriatic retinal images were captured by trained field operators. Two ophthalmology fellows provided robust and reliable labels for model training using a pragmatic definition of gradability. The requirement for whole optic disc capture within retinal images was included in the gradability definitions given its clinical significance in DR. Despite the more challenging retinal image dataset, inter-grader agreement was good with excellent intra-grader reliability. We applied up-to-date, computationally efficient models (EfficientNet) to maximise utility within mobile and portable retinal imaging devices and achieved competitive performance with minimal pre-processing. Study limitations are the lack of an external dataset of handheld, non-mydriatic retinal images for additional validation. Quantitative data on why images were deemed ungradable by individual graders were not available. However, graders subjectively reported that obscuration was the most common issue affecting ungradable images, followed by blurring and an incompletely captured optic disc in smaller number of images. Future studies should develop field detection models which can be combined with gradability models. Prospective clinical validation studies of handheld retinal imaging devices should ascertain effects on STDR detection.

## Supplementary Information

Supplementary Information.

## Data Availability

Researchers can apply to Moorfields Research Management Committee for access to the image and numerical study data for use in an ethics approved project by emailing moorfields.resadmin@nhs.net.

## Code availability

Models were developed using standard open-source python libraries and TensorFlow 2.1 (https://www.tensorflow.org).
