# Supplementary material for: Deep learning for gradability classification of handheld, non-mydriatic retinal images
Source: Sci Rep. 2021 May 4;11:9469. doi: 10.1038/s41598-021-89027-4 (PMC8096843; doi:10.1038/s41598-021-89027-4)
Supplement: Supplementary file 1 — Supplementary Information. [file 41598_2021_89027_MOESM1_ESM.docx]

# Supplementary Information

## Deep Learning for Gradability Classification of Handheld, Non-mydriatic Retinal Images

Paul Nderitu; Joan M. Nunez do Rio; Rajna Rasheed; Rajiv Raman; Ramachandran Rajalakshmi; Christos Bergeles; Sobha Sivaprasad; for the SMART India study group.

## Supplementary Figure 1. Compact Model (EfficientNet-B0) Architecture


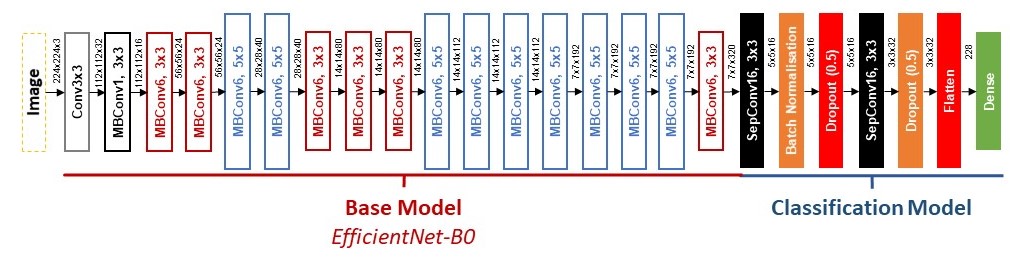


Conv: Convolutional layer, MBConv: Mobile inverted bottleneck convolution block, SepConv: Depth-wise separable convolution layer

## Supplementary Figure 2. Large Model (EfficientNet-B5) Architecture


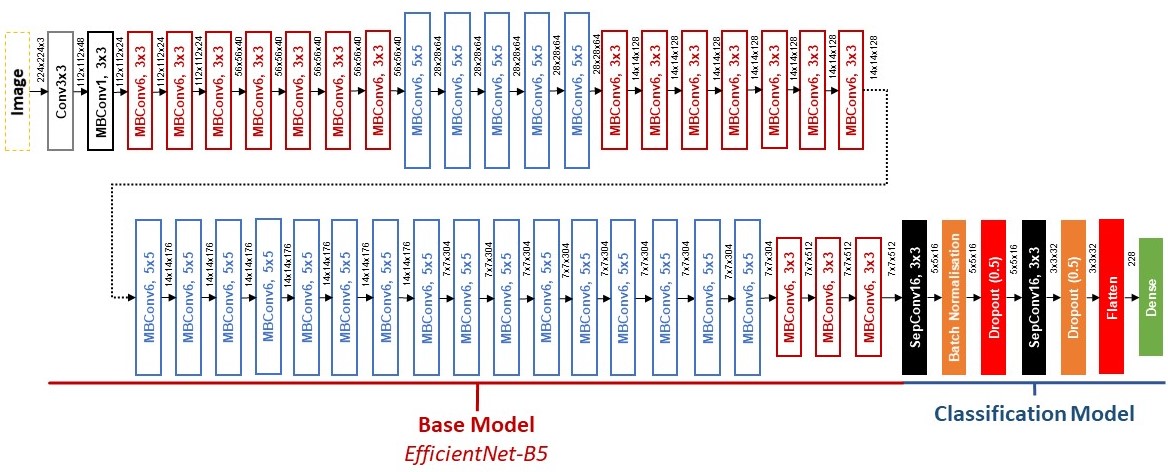


Conv: Convolutional layer, MBConv: Mobile inverted bottleneck convolution block, SepConv: Depth-wise separable convolution layer

## Supplementary Figure 3. Large Model (EfficientNet-B5) Gradability ROC and PR Curves


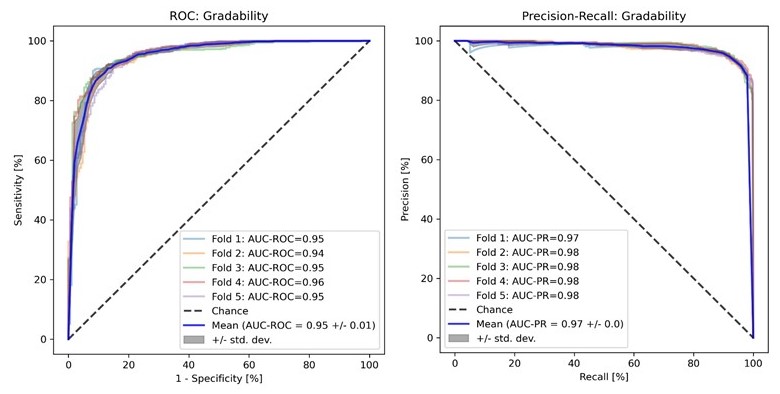


ROC: Receiver operating characteristic, AUC-ROC: Area under the receiver operating characteristic curve, AUC-PR: Area under the precision recall curve, std. dev: Standard deviation

## Supplementary Table 1. Large Model (EfficientNet-B5) and Grader Performance

|  | OP  Threshold | Gradability | Grader 1 | | Total  N (%) | Precision  [Recall] | Kappa  (SE) |
| --- | --- | --- | --- | --- | --- | --- | --- |
|  |  |  | **Ungradable**  N (%) | **Gradable**  N (%) |  |  |  |
| Efficient  Net-B5  Model | **OP1/2**  0.5 | **Ungradable** | **738 (22.6)** | 341 (10.5) | 1,079 (33.1) | 0.68 [0.89] | **0.69** |
|  |  | **Gradable** | 87 (2.7) | **2,095 (64.2)** | **2,182 (66.9)** | **0.96 [0.86]** | (0.01) |
|  |  | | | | | | |
|  | **OP3**  0.33 | **Ungradable** | **657 (20.1)** | 168 (5.2) | 825 (25.3) | 0.80 [0.80] | **0.73** |
|  |  | **Gradable** | 168 (5.2) | **2268 (69.5)** | **2,436 (74.7)** | **0.93 [0.93]** | (0.01) |
|  |  |  |  |  |  |  |  |
| Grader 2 | N/A | **Ungradable** | **544 (16.7)** | 215 (6.6) | 759 (23.3) | 0.72 [0.66] | **0.59** |
|  |  | **Gradable** | 281 (8.6) | **2,221 (68.1)** | **2,502 (76.7)** | **0.89 [0.91]** | (0.02) |
|  |  | | | | | | |
| Total N (%) | | | 825 (25.3) | **2,436 (74.7)** | 3,261 (100) | N/A | |

OP: Operating point, SE: Standard error. Operating point 1 and 2 had a coincident threshold value of 0.5.
